# Document Retrieval for Precision Medicine Using a Deep Learning Ensemble Method

**DOI:** 10.2196/28272

**Published:** 2021-06-29

**Authors:** Zhiqiang Liu, Jingkun Feng, Zhihao Yang, Lei Wang

**Affiliations:** 1 College of Computer Science and Technology Dalian University of Technology Dalian China; 2 Beijing Institute of Health Administration and Medical Information Beijing China

**Keywords:** biomedical information retrieval, document ranking, precision medicine, deep learning

## Abstract

**Background:**

With the development of biomedicine, the number of biomedical documents has increased rapidly bringing a great challenge for researchers trying to retrieve the information they need. Information retrieval aims to meet this challenge by searching relevant documents from abundant documents based on the given query. However, sometimes the relevance of search results needs to be evaluated from multiple aspects in specific retrieval tasks, thereby increasing the difficulty of biomedical information retrieval.

**Objective:**

This study aimed to find a more systematic method for retrieving relevant scientific literature for a given patient.

**Methods:**

In the initial retrieval stage, we supplemented query terms through query expansion strategies and applied query boosting to obtain an initial ranking list of relevant documents. In the re-ranking phase, we employed a text classification model and relevance matching model to evaluate documents from different dimensions and then combined the outputs through logistic regression to re-rank all the documents from the initial ranking list.

**Results:**

The proposed ensemble method contributed to the improvement of biomedical retrieval performance. Compared with the existing deep learning–based methods, experimental results showed that our method achieved state-of-the-art performance on the data collection provided by the Text Retrieval Conference 2019 Precision Medicine Track.

**Conclusions:**

In this paper, we proposed a novel ensemble method based on deep learning. As shown in the experiments, the strategies we used in the initial retrieval phase such as query expansion and query boosting are effective. The application of the text classification model and relevance matching model better captured semantic context information and improved retrieval performance.

## Introduction

In recent years, biomedical research has developed rapidly leading to a great increase in the number of biomedical publications. Biomedical development promotes the treatment of intractable diseases; however, the huge number of biomedical documents brings a great challenge for researchers in obtaining the documents related to one topic. Biomedical information retrieval (IR) is thus a hot research topic in the biomedical domain.

Given a query, biomedical IR systems are designed to provide users with all relevant documents in a ranked list, sorted according to their relevance to the query. The relevance can be evaluated by applying different IR models [1-4] based on either the occurrence of query terms in the documents or probabilistic measures. However, it is difficult to achieve an ideal retrieval performance when directly applying these IR models to biomedical IR. One possible reason is that the IR models cannot interpret the semantic information of the query and can only use frequencies and other features of query terms appearing in documents to determine the relevance. For example, when given a query “How is melanoma treated?” the goal of the query is to find relevant documents focusing on the treatment of melanoma. Since some documents focusing on other aspects such as clinical trials and pathology also contain many instances of the query term melanoma, the model considers these documents related, thus leading a poor retrieval performance. Moreover, biomedical documents usually contain diversified concept expressions and abundant professional vocabularies, and these vocabularies can usually be replaced by their synonyms or abbreviations, which increases the difficulty in relevance evaluation. In addition, in some specific biomedical IR tasks, the relevance between query and document needs to be evaluated from multiple aspects. For example, in the precision medicine (PM) retrieval task, for patients with certain diseases and genetic variants, researchers need to connect patients with experimental treatments if existing treatments have been ineffective; the retrieval goal is to find the experimental treatments for which the patients are eligible. The retrieval system must determine whether the patient meets the experiment requirements from multiple aspects such as disease, genetic variants, age, and so on, thereby increasing the difficulty and cost of system design. All the above situations bring domain-specific challenges for biomedical IR. Therefore, it is necessary to explore an effective biomedical IR method.

To alleviate the above problems, in this paper we propose a novel ensemble method based on deep learning for biomedical IR. Given the patient’s disease, genetic variants, and demographic information, our method aims to find documents that provide information relevant to the treatment of the patient’s disease. Therefore, our method needs to evaluate documents from treatment, disease, and gene dimensions. In particular, existing studies have proved that the IR task can be treated as a relevance matching problem between query and document [5,6]. Based on this, researchers have proposed a variety of matching models from different perspectives. To refine the retrieval performance in these approaches, after obtaining an initial ranked list of relevant documents retrieved through a search engine, a relevance matching model is deployed as a re-ranker over the ranked list to re-rank all relevant documents. Following other researchers, we also consider the relevance matching model as a component of the re-ranker to re-rank relevant documents. Specifically, our method can be divided into two phases: initial retrieval and re-ranking. During the initial retrieval phase, to alleviate the problem of diverse concept expressions and abundant professional vocabularies with synonyms in biomedical IR, we introduce external biomedical resources to create a local database, and based on this, we design effective query expansion strategies to reformulate the original query by supplementing relevant terms to better describe the retrieval need. We also design query boosting strategies to adjust the weights of query terms. During the re-ranking phase, to alleviate the problem of a retrieval model that cannot interpret the query semantics, we employ a relevance matching model based on deep learning to capture semantic signals between query and document from the disease dimension. To make our re-ranker evaluate articles from multiple dimensions, we built an effective text classification model to determine whether a document is treatment-focused. In particular, to combine the two models effectively, we apply a logistic regression (LR) model to output the final score for each document and reorder our initial ranking list according to their scores. Experimental results on the collections from the Text Retrieval Conference (TREC) 2019 PM track demonstrate that the proposed method can effectively improve the retrieval performance in biomedical IR.

We summarize the contributions of our work as follows:

- We propose an ensemble method based on deep learning to evaluate relevance between query and document from multiple dimensions in biomedical IR.
- We introduce an effective relevance matching model and text classification model to fully capture semantic information from a query and refine the retrieval performance in biomedical IR.
- We apply the LR method to combine the relevance matching model and text classification model, and experimental results show that it is more effective than the voting method.

The remainder of this paper is organized as follows. In section 2, we discuss some related work. In section 3, we describe our method in detail. In section 4, we discuss the experiments conducted to evaluate the effectiveness of the proposed method. In section 5, we conclude the paper and provide suggestions for future work.

## Methods

### Model Architecture

**Figure 1:**
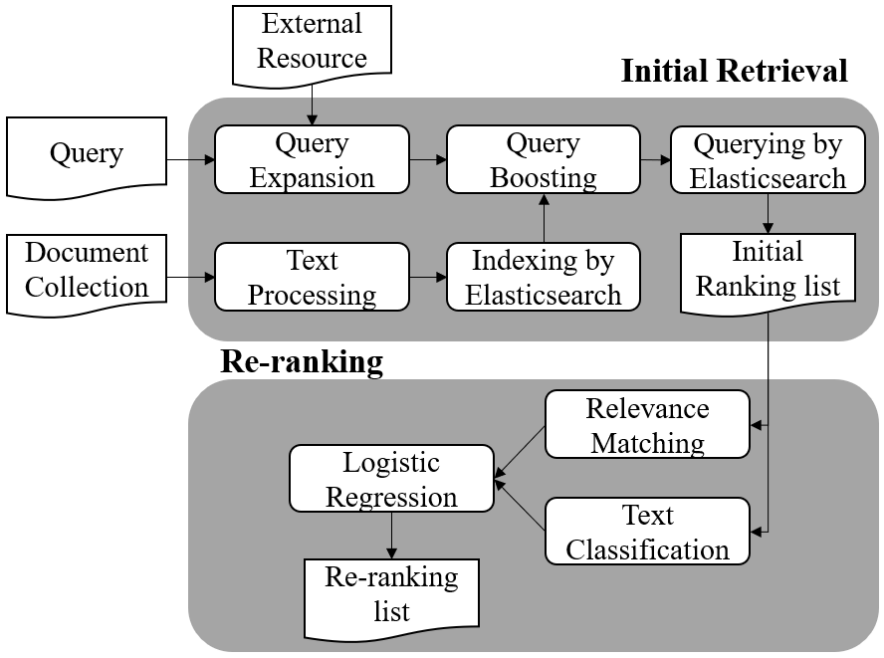
Overview of the architecture of our method.

In this section, we illustrated the framework of our ensemble method and provided detailed descriptions. Figure 1 describes the overview of the architecture of our method, divided into two phases: initial retrieval phase and re-ranking phase. In the initial retrieval phase, we first supplemented query terms through query expansion strategies we designed, then we used a search engine named Elasticsearch to index documents, and finally, we applied query boosting to obtain an initial ranked list of relevant documents. In the re-ranking phase, we first employed a text classification model and relevance matching model to evaluate documents respectively from different dimensions, then we combined their outputs through LR, and finally we re-ranked all the documents from the initial ranking list according to their relevance scores.

#### Initial Retrieval

Given a query Q = {*q_1_*, *q_2_*, ..., *q_M_*}, the goal of this phase is to obtain an initial ranking list D = {*d_1_*, *d_2_*, …, *d_N_*}, where *q_i_* represents the i-th term in the query, *d_i_* represents a candidate document related to query, M stands for the number of query terms, and N stands for the number of candidate documents. Specifically, we chose BM25 [7], a probabilistic retrieval model commonly used in search engines, to calculate the relevance score between query and document. To make our retrieval process more efficient and convenient, after preprocessing the whole document collection, we used Elasticsearch, an open-source Lucene-based full-text search engine, to index all documents and search relevant candidate documents.

#### Text Processing

The document collection is a snapshot of PubMed abstracts, and XML and TXT versions are available. The XML versions have the complete information for each abstract. We extracted text information from fields that might be useful like ArticleTitle, Abstract, ChemicalList, MeshHeadingList, and OtherAbstract fields. The information was saved in JSON format, which is convenient for index building.

#### Query Expansion

Considering that biomedical documents usually contain abundant specialized words with synonyms and abbreviations, we first built a local database to introduce external biomedical resources to improve the recall rate of retrieval results. In the database, we stored biomedical disease and gene entities, as well as their entity IDs, synonyms, hypernyms, and acronyms. In particular, the disease information is derived from the Comparative Toxicogenomics Database [8], while the genetic information is derived from the National Center for Biotechnology Information gene database. Next, we supplemented query terms with their synonyms and acronyms to better describe the retrieval need. Since our method aims to retrieve documents that focus on disease treatment, we additionally introduced some treatment-related keywords into the queries such as surgery, therapy, patient, resistance, recurrence, therapeutic, prevent, prophylaxis, prophylactic, prognosis, outcome, survival, treatment, and efficacy.

#### Query Boosting

To improve the retrieval performance in the initial retrieval phase, we used query boosting to define different weights for different query fields during the retrieval process. Specifically, we compiled the query template provided by Elasticsearch to boost some query fields. In our custom query template, there were 4 query fields: disease, genetic variant, treatment keyword, and demographic. Among them, disease and gene fields were considered as the most important query fields, hence they had higher weight values than other fields.

#### Querying

For each original query Q, we reformulated their query terms through query expansion and defined their weights by query boosting. Then we used Elasticsearch based on BM25 to retrieve candidate documents related to Q and rank them in descending order according to their BM25 scores. Finally, we obtained our initial ranking list D.

In this phase, the relevance scores of documents were calculated based on the prominence of query terms appearing in documents, and the semantic information was not considered. In the next phase, all candidate documents in D were reevaluated from multiple dimensions.

#### Re-Ranking

The goal of the re-ranking phase was to refine the retrieval performance by re-ranking all documents obtained from the previous phase. Given the initial ranking list D = {*d_1_*, *d_2_*, …, *d_N_*}, our re-ranker reevaluated relevance between queries and documents from multiple dimensions and reordered them in descending order according to their new relevance scores.

During the re-ranking phase, for all documents in the initial ranking list, a text classification model and relevance matching model were employed to reevaluate these documents from different dimensions. Then, an LR model was applied to combine the two models and output final relevance scores. Finally, according to the final scores, a heuristic rule was applied to re-rank these documents.

#### Text Classification

In our method, a text classification model was used to determine whether a document was treatment-focused. As a component of the re-ranker, our text classification model is a binary classification model.

When preprocessing the documents in initial ranking list D, for each document, we connected its title and abstract with delimiter SEP, and converted all letters to lowercase. Next, we replaced all numbers that appeared in the document with token NUM. Finally, we normalized it by defining the maximum document length h: if the document is shorter than h, we used token PAD filling it to h; otherwise, we truncated it to h directly.

**Figure 2:**
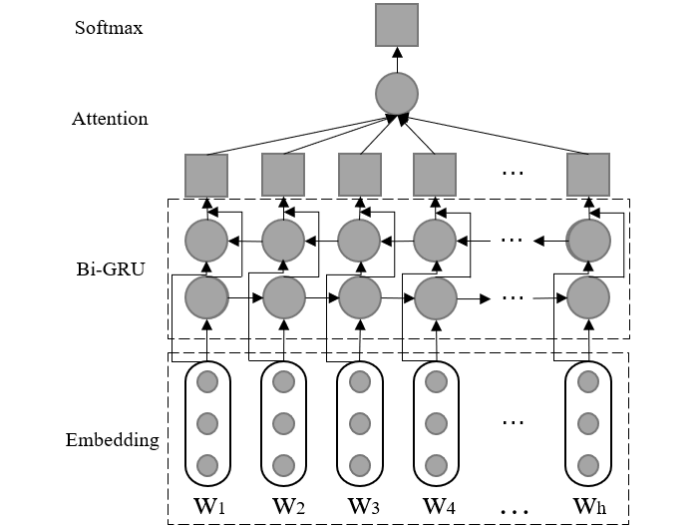
Structure of the text classification model.

When building the text classification model (bidirectional gated recurrent unit– attention [BiGRU-Att]), we adopted a bidirectional gated recurrent unit (GRU) [9] layer to encode the input word sequence and capture the context information. Also, an attention mechanism [10] was used to focus on relevant words to each category so that our method accurately picked out the corresponding documents to a query. We illustrate the structure of our text classification model in Figure 2.

Given a document *d* = {*w_1_*, *w_2_*, …, *w_h_*}, where *w_i_* represents the i-th word in it, the Embedding layer represented it to the word embedding matrix *M* = {*v_1_*, *v_2_*, …, *v_h_*}, where *v_i_* represented the embedding vector of the i-th word.

In the bidirectional GRU layer, given the input *v_t_*, the hidden state *h_t_* was computed as follows:


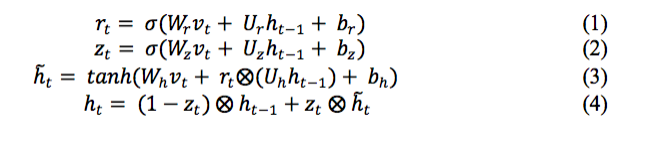


where, *r_t_* is the reset gate, *z_t_* is the update gate, *h_t–1_* is the previous state, *h ~_t_* is the candidate state at time t, *v_t_* is the sequence vector at time t, σ(·) and tanh(·) is sigmoid and hyperbolic tangent functions. *b_z_*, *b_r_*, and *b_h_* are bias terms. The operator 
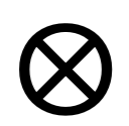
 denotes element-wise multiplication.

To generate the context feature matrix *H*, we concatenated the matrixes *H_forward_* and *H_back_*, which are the output of the forward and back GRU’s hidden layers respectively, namely, *H* = [*H_forward_*; *H_back_*].

In the Attention layer, for the input xt, the attention weight αt was calculated as follows:


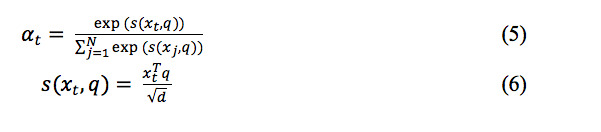


where, d is the dimension of input vector, and q is the query vector. Finally, the probability that the input document was classified into each category was calculated through a softmax layer.

#### Relevance Matching

In our method, a relevance matching model was applied to reevaluate the relevance between query and document from disease dimension.

When preprocessing the documents in initial ranking list D, for each document, based on the text classification preprocessing, we removed all stop words in the document and applied the Porter Stemmer [11] to stem the remain words. Since the biomedical documents usually contain abundant professional words with synonyms, for all disease entities in the document, if their synonyms appear in the same document, we replaced them with the same entity ID. Simultaneously, we also used the same entity ID to replace the disease entities that belonged to the same concept in the query.

To consider the semantic information between the query and the document when reevaluating the relevance, we adopted the MatchPyramid [12] model as our relevance matching model. Specifically, given the query q and the document d, MatchPyramid takes the query-document pair (*q*, *d*) as input. Then, a dot product operation as follows was employed to generate a matching matrix S between query and document.


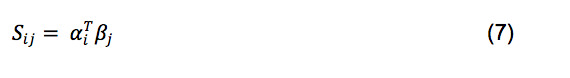


Where α_i_ and β_j_ are the i-th and the j-th word embedding vectors from *q* and *d*, respectively. Finally, hierarchical convolutional neural networks and multilayer perceptron were applied to output the relevance score.

#### Logistic Regression

In our method, given the query q, each document d in the initial ranking list was judged with a simple measure: definitely relevant, partially relevant, and not relevant. Therefore, the goal of employing the LR model was to determine the relevance levels of documents by comprehensively considering the evaluation results of all models.

When building our LR model, we transferred the problem of relevance evaluation into a classification problem. Namely, given the labels of definitely relevant, partially relevant, and not relevant for each query, we used the LR model to determine the label of each candidate document. Since ordinary LR can only handle binary classification problems, we built 3 binary classification models (LR1, LR2, and LR3) based on the LR (ie, our LR model [LR] is composed of the 3 binary classifiers). In LR1, definitely relevant documents were treated as positive samples while other documents were treated as negative samples. In LR2, partially relevant documents were treated as positive samples while other documents were treated as negative samples. In LR3, documents that were not relevant were treated as positive samples while other documents were treated as negative samples.

Specifically, for a document d from the initial ranking list, we built 3 LR models with [*v_1_*, *v_2_*, *v_1_^2^*, *v_2_^2^*] as input and the sigmoid function as the activation function. Cross entropy was used as the loss function. *v_1_* was the probability that measure d is treatment-focused or not, and *v_2_* was the relevance score. We obtained the 2 values from the text classification model and the relevance matching model. Then we computed their outputs that represented probabilities on the 3 labels, respectively. Finally, we chose the label that indicated the maximum probability as the final output of the LR model.

#### Re-Ranking Rule

After determining the relevance level of each document through the LR model, we used a heuristic rule to reorder the initial ranking list. Specifically, we ranked the definitely relevant documents over partially relevant documents, and the documents that were not relevant were ranked last. For the documents belonging to the same relevance level, we ranked them in descending order according to their BM25 scores obtained through Elasticsearch.

## Results

### Experiment Settings

TREC 2019 PM Track focused on an important use case in PM for clinical decision support: providing useful PM-related information to clinicians treating cancer patients. Participants of the track were challenged with retrieving (1) biomedical articles in the form of article abstracts (largely from MEDLINE/PubMed) addressing relevant treatments for the given patient and (2) clinical trials (from ClinicalTrials.gov) addressing relevant clinical trials for which the patient was eligible. In particular, we mainly focused on the first task. The first task aimed to retrieve relevant treatment information for the given diseases from the scientific literature. This task provided 40 topics consisting of the disease, genetic variants, and demographic information about the patients. And for each topic, the participating system needed to return up to 1000 related documents retrieved from the scientific literature. For judging the relevance of documents, the organizer mainly evaluated from 3 dimensions: treatment, disease, and gene. To evaluate each retrieval result, precision at rank 10 (P@10), R-precision (R-prec), and inferred normalized discounted cumulative gain (infNDCG) are used as the evaluation metrics [13].

Specifically, we used TREC-Eval, a tool provided by the TREC organizer, to implement the evaluation of our experimental results on the 3 metrics. To train the BiGRU-Att, we used the gold standard of the TREC 2017 PM Track [14] as the data source of the model. According to the annotation of the gold standard, we first labeled all documents with 2 categories: treatment-focused or not. Then, we randomly divided the whole dataset into a training set, a development set, and a test set at a ratio of 8:1:1. When training the model, we applied the Adam algorithm [15] for parameter optimization and used the development set to optimize the hyperparameters. Finally, we applied the early stop mechanism to select the number of training iterations. Table 1 lists the hyperparameters of the BiGRU-Att. When the training was complete for each document in the initial ranking list, we used BiGRU-Att to determine whether it was treatment-focused.

To train the MatchPyramid, we also used the gold standard of the TREC 2017 PM Track as the data source of the model. For each document in the gold standard, according to its annotation on the disease dimension, we adopted the following labeling strategies: if the disease was exact, we labeled it with 2; if the disease was more specific or more general, we labeled it with 1; and if the disease is not disease, we labeled it with 0. Then, we randomly divided all data into a training set, development set, and test set at a ratio of 8:1:1. To implement the model, we used MatchZoo [16], an open source text matching tool, to build the MatchPyramid. When training the model, we applied the Adagrad algorithm [17] for parameter optimization and used the development set to optimize the hyperparameters. Finally, we applied the early stop mechanism to select the number of training iterations. Table 1 lists the hyperparameters of the MatchPyramid. When the training was complete for each document in the initial ranking list, we used MatchPyramid to determine its relevance level on the disease dimension.

To train the LR1, LR2, and LR3, the gold standard of the TREC 2017 PM was used as the data source of the models. According to the relevance level annotated in the gold standard, we adopted the following labeling strategies to construct a dataset for each LR model: given a topic in LR1, we labeled definitely relevant documents with 1 and others with 0; in LR2, we labeled partially relevant documents with 1 and others with 0; and in LR3, we labeled unrelated documents with 1 and others with 0. We then randomly divided each dataset into a training set, development set, and test set at a ratio of 8:1:1. When training these models, we used scikit-learn [18], a machine learning library, to build and select the 3 LR models, and we applied the early stop mechanism to select the number of training iterations. When the training was complete for each document in the initial ranking list, we used the 3 LR models in turn to predict its relevance level and re-ranked the initial ranked list according to our heuristic rule.

**Table 1:** The hyperparameters of BiGRU-Att and MatchPyramid.

Model and parameter		Value
**BiGRU-Att^a^**		
	Document max length	256
	Word embedding dimension	200
	Hidden layer dimension	200
	Learning rate	0.001
	Batch size	128
**MatchPyramid**		
	Query max length	30
	Document max length	200
	Word embedding dimension	200
	Number of convolution kernel	400
	Convolution kernel size	5
	Learning rate	0.001
	Batch size	64

^a^BiGRU-Att: bidirectional gated recurrent unit– attention.

### Query Expansion Experiment

To explore the impact of query expansion on retrieval performance, we conducted corresponding experiments with different expansion strategies. As shown in Table 2, when expanding the disease field, using synonym expansion achieved better retrieval performance while using hypernym expansion made the retrieval performance worse. The reason is that hypernyms represent a more general concept, but the TREC PM task requires retrieving the treatment information about a specific disease. Therefore, the disease name itself should be paid more attention during the retrieval process, which made synonym expansion outperform hypernym expansion. When expanding the gene field, synonym expansion greatly reduced the search performance, and compared with not using query expansion, acronym expansion had no obvious impact on retrieval performance. By analyzing retrieval results, we found that the treatment-focused articles usually did not mention related genes. After synonym expansion for gene, the proportion of gene keywords in query terms increased sharply, so when we searched based on exact matching models such as BM25, genetics-focused articles obtained a higher score, leading to a poor performance. In addition, the gene entities were usually expressed in abbreviation form, and their acronyms were rarely used. Therefore, acronym expansion had little effect on search results. Finally, it can be seen from Table 2 that, after treatment keywords were added, the treatment-focused documents achieved higher scores leading to improved retrieval performance.

In subsequent experiments, we adopted the following query expansion strategy: for the disease field, we used synonym expansion, and for the gene field, we did not. In addition, we added a treatment field to supplement treatment-related keywords.

**Table 2:** Experimental results of query expansion.

Disease		Gene		Treatment	P@10^a^	R-prec^b^	infNDCG^c^
Syn^d^	Hyper^e^	Syn	Acro^f^				
—^g^	—	—	—	—	0.5325	0.2934	0.4585
✓	—	—	—	—	0.5675	0.3113	0.4783
—	✓	—	—	—	0.5300	0.2942	0.4577
—	—	✓	—	—	0.4550	0.2801	0.4324
—	—	—	✓	—	0.5325	0.2933	0.4580
—	—	—	—	✓	0.5425	0.3104	0.4732
✓	—	—	—	✓	0.5700	0.3223	0.4882

^a^P@10: precision at rank 10.

^b^R-prec: R-precision.

^c^infNDCG: inferred normalized discounter cumulative gain.

^d^Syn: synonym.

^e^Hyper: hypernym.

^f^Acro: acronym.

^g^No expansion.

^h^Corresponding term is not applied for expansion.

### Query Boosting Experiment

In our method, we used query boosting to optimize the weights of different query fields. Our query template included query clauses for disease, gene, treatment, and demographic information about the patients, respectively, and they are expressed as Q_d_, Q_g_, Q_t_, and Q_p_. To enhance the performance during the initial retrieval phase, we conducted the corresponding experiment by setting different weights for different fields. The experimental results are shown in Table 3. Among them, when we boost the weight of a field, the weights of other fields are set to 1.0.

It can be seen from Table 3 that when we boosted the weights of Q_d_ and Q_g_, the retrieval performance improved, indicating that disease and genetic variants are more important than other clauses. When the weights of Q_t_ and Q_p_ are boosted, the retrieval performance was not improved, indicating that the treatment keywords and demographic of patients cannot provide more specific information for retrieval.

In subsequent experiments, we adopted the following query boosting strategy: Q_d_ = 1.5, Q_g_ = 1.5, Q_t_ = 1.0 and Q_p_ = 1.0.

**Table 3:** Experimental results of query boosting.

Strategy	P@10^a^	R-prec^b^	infNDCG^c^
No boosting	0.5700	0.3223	0.4882
Q_d_ = 1.5	0.5750	0.3246	0.4923
Q_g_ = 1.5	0.5750	0.3238	0.4911
Q_t_ = 1.5	0.5700	0.3231	0.4893
Q_p_ = 1.5	0.5700	0.3225	0.4884
Q_d_ = 1.5, Q_g_ = 1.5	0.5800	0.3250	0.4981

^a^P@10: precision at rank 10.

^b^R-prec: R-precision.

^c^infNDCG: inferred normalized discounter cumulative gain.

### Ensemble Experiment

To explore the impact of models applied in the re-ranking phase on retrieval performance, we conducted the ensemble experiment. Based on the initial retrieval phase, we combined one model at a time to reorder the documents in the initial ranking list. To explore the effectiveness of the LR on integrating BiGRU-Att and MatchPyramid, we used a voting algorithm for comparison. That is, for one document, if it was predicted as treatment-focused and its relevance score was greater than zero, then it was definitely relevant; if it was predicted as not treatment-focused and its relevance score was less than zero, then it was not relevant; otherwise, it was partially relevant. The experimental results are shown in Table 4. Among them, the baseline is the performance of the initial retrieval phase, and Ensemble-TC and Ensemble-RM denote adding BiGRU-Att and MatchPyramid, respectively, ALL-Voting and ALL-LR denote integrating all models with voting algorithm and LR, respectively.

It can be seen from Table 4 that when integrating the BiGRU-Att, P@10 is increased from 0.58 to 0.63. This is because BiGRU-Att can determine whether a document is treatment-focused, and rank these documents in a higher position. When integrating the MatchPyramid, the retrieval performance is also improved due to the ability of our deep matching model on capturing semantic context information between query and document. Also, when integrating by the LR, the retrieval performance is better than the voting algorithm, and this suggests that the application of LR to ensemble models is more effective than the voting algorithm.

**Table 4:** Ensemble experiment results.

Model	P@10^a^	R-prec^b^	infNDCG^c^
Baseline	0.5800	0.3250	0.4981
Ensemble-TC^d^	0.6300	0.3324	0.5142
Ensemble-RM^e^	0.6075	0.3393	0.5049
ALL-Voting^f^	0.6375	0.3348	0.5143
ALL-LR^g^	0.6500	0.3391	0.5237

^a^P@10: precision at rank 10.

^b^R-prec: R-precision.

^c^infNDCG: inferred normalized discounter cumulative gain.

^d^Ensemble-TC: baseline + BiGRU-Att.

^e^Ensemble-RM: baseline + MatchPyramid.

^f^ALL-Voting: all models integrated with voting algorithm.

^g^ALL-LR: all models integrated with LR.

### Performance Comparison Experiment

To explore the performance of the proposed method on the biomedical IR task, we compared our method with other deep learning–based systems participating in TREC 2019 PM task. The experimental results are shown in Table 5.

**Table 5:** Performance comparison of various methods.

Team	Method	P@10^a^	R-prec^b^	infNDCG^c^
Ours	baseline	0.5800	0.3250	0.4981
Ours	ALL-LR^d^	0.6500	0.3391	0.5237
CCNL^e^	SciBERT	0.6500	0.3066	0.5309
DUTIR^f^	Ensemble	0.5975	0.3273	0.5108
ECUN_ICA^g^	Doc2vec	0.5675	0.2718	0.4672

^a^P@10: precision at rank 10.

^b^R-prec: R-precision.

^c^infNDCG: inferred normalized discounter cumulative gain.

^d^ALL-LR: all models integrated with LR.

^e^CCNL: team name.

^f^DUTIR: team name.

^g^ECUN_ICA: team name.

Among these comparison systems based on deep learning, CCNL treated the document re-ranking problem as a two-category problem (ie, the documents definitely relevant and partially relevant to given topics are considered positive samples while unrelated documents are considered negative samples). Team CCNL trained a SciBERT (Scientific Bidirectional Encoder Representations from Transformers) [19] to classify all documents. DUTIR is the system we submitted in the task. In this system, we combined recurrent convolutional neural networks for text classification [20], deep relevance matching model for ad-hoc retrieval, and recurrent convolutional neural networks for text classification [21] to evaluate candidate documents from treatment, disease, and gene dimensions, respectively. Moreover, ECNU_ICA trained a doc2vec model to encode both documents and queries into fixed-length vectors and then used their cosine scores as similarity metrics.

As can be seen from Table 5, compared with other methods, the R-prec of our method (ALL-LR) was the best (0.3391). Additionally, our R-prec during the initial retrieval phase reached 0.3250, which was better than most of the other methods. This indicates that our query expansion and query boosting strategies worked well on biomedical IR. After integrating our re-ranker models, our R-prec further improved from 0.3250 to 0.3391. Meanwhile, our P@10 improved from 0.58 to 0.65, which was the same as the best result of other methods. Moreover, the result of infNDCG improved from 0.4981 to 0.5237. This shows that during the re-ranking phase, the semantic context features between queries and documents were better captured, thereby optimizing the initial retrieval performance. However, although the result of infNDCG improved, it was still lower than that of CCNL. One possible reason is that our ensemble method inevitably introduced the problem of error propagation because the accuracies of the employed models were not 100% and the deviation of these models led to some mistakes in determining the relevance level of some documents.

## Discussion

### Principal Findings

In this paper, we proposed a novel ensemble method based on deep learning for biomedical IR. The experimental results showed that (1) the query expansion and query boosting strategies we designed are effective, (2) the application of the text classification model and relevance matching model fully captured semantic context information and improved the retrieval performance, (3) using LR to combine models was more effective than the voting algorithm, and (4) our ensemble method evaluated relevance between query and document from multiple aspects in biomedical IR.

### Limitations

However, there is still much room for performance improvement. The problem of error propagation limits the performance of the ensemble method, and using a joint model to address the problem may be an effective solution. In addition, domain feature engineering has been proven to effectively improve retrieval performance, and, therefore, constructing the domain features to enhance the retrieval performance is also our future work.

### Conclusion

In this work, we introduced the ensemble method for the relevance evaluation from multiple aspects in biomedical IR. Our method annotated the usefulness of query expansion and query boosting by simultaneously applying them to obtain the large number of documents related to the query. To evaluate relevance from multiple dimensions and refine the retrieval performance, we integrated the text classification model and relevance matching model through LR modelling. Overall, we attributed the improvement of the proposed method in biomedical IR to two aspects: initial retrieval strategies and re-ranking models. For the initial retrieval strategies, we expanded the query terms with their synonyms and defined different weights for different query fields, which improved the accuracy and recall rate during the initial retrieval phase. For the re-ranking models, we introduced the text classification model and relevance matching model, which evaluated the relevance of search results from multiple dimensions. These aspects jointly contributed to improvement in retrieval performance, and the proposed method showed the effectiveness of evaluating relevance from multiple aspects.

## Abbreviations

BiGRU-Att: bidirectional gated recurrent unit– attention
GRU: gated recurrent unit
infNDCG: inferred normalized discounter cumulative gain
IR: information retrieval
LR: logistic regression
P@10: precision at rank 10
PM: precision medicine
R-prec: R-precision
SciBERT: Scientific Bidirectional Encoder Representations from Transformers
TREC: Text Retrieval Conference
